# Pathway Based Analysis of Mutation Data Is Efficient for Scoring Target Cancer Drugs

**DOI:** 10.3389/fphar.2019.00001

**Published:** 2019-01-23

**Authors:** Marianna A. Zolotovskaia, Maxim I. Sorokin, Anna A. Emelianova, Nikolay M. Borisov, Denis V. Kuzmin, Pieter Borger, Andrew V. Garazha, Anton A. Buzdin

**Affiliations:** ^1^Oncobox Ltd., Moscow, Russia; ^2^Department of Oncology, Hematology and Radiotherapy of Pediatric Faculty, Pirogov Russian National Research Medical University, Moscow, Russia; ^3^The Laboratory of Clinical Bioinformatics, IM Sechenov First Moscow State Medical University, Moscow, Russia; ^4^Omicsway Corp., Walnut, CA, United States; ^5^Science-Educational Center Department, M. M. Shemyakin and Yu. A. Ovchinnikov Institute of Bioorganic Chemistry, Russian Academy of Sciences, Moscow, Russia; ^6^Laboratory of the Swiss Hepato-Pancreato-Biliary, Department of Surgery, Transplantation Center, University Hospital Zurich, Zurich, Switzerland

**Keywords:** cancer, DNA mutation, molecular pathways, biomarker, target drugs, tyrosine kinase inhibitors, nibs, mabs

## Abstract

Despite the significant achievements in chemotherapy, cancer remains one of the leading causes of death. Target therapy revolutionized this field, but efficiencies of target drugs show dramatic variation among individual patients. Personalization of target therapies remains, therefore, a challenge in oncology. Here, we proposed molecular pathway-based algorithm for scoring of target drugs using high throughput mutation data to personalize their clinical efficacies. This algorithm was validated on 3,800 exome mutation profiles from The Cancer Genome Atlas (TCGA) project for 128 target drugs. The output values termed Mutational Drug Scores (MDS) showed positive correlation with the published drug efficiencies in clinical trials. We also used MDS approach to simulate all known protein coding genes as the putative drug targets. The model used was built on the basis of 18,273 mutation profiles from COSMIC database for eight cancer types. We found that the MDS algorithm-predicted hits frequently coincide with those already used as targets of the existing cancer drugs, but several novel candidates can be considered promising for further developments. Our results evidence that the MDS is applicable to ranking of anticancer drugs and can be applied for the identification of novel molecular targets.

## Introduction

Globally, cancer is one of the major causes of death (Centers for Disease Control and Prevention, 2017). For several decades, chemotherapy remains a key treatment for many cancers, often with impressive success rates. For example, its use in testicular cancer turned near complete mortality to >90% disease-specific survival (Hanna and Einhorn, 2014; Oldenburg et al., 2015). However, most of the advanced cancers remain incurable and/or unresponsive using standard chemotherapy approaches, frequently develop resistance to treatments and relapse (Vasey, 2003; Housman et al., 2014). More recently, a new generation of drugs has been developed that specifically target functional tumor marker molecules. These medicines termed *Target drugs* have one or a few specific molecular targets in a cell (Druker et al., 2001a,b; Sawyers, 2004; Spirin et al., 2017). They have greater selectivity and generally lower toxicity than the conventional chemotherapy (Joo et al., 2013). Structurally, they can be either low molecular mass inhibitor molecules or monoclonal antibodies (Padma, 2015). The repertoire of their molecular targets is permanently growing and now includes receptor and intracellular tyrosine kinases (Baselga, 2006), vascular endothelial growth factor (Rini, 2009), immune checkpoint molecules such as PD1, PDL1, and CTLA4 (Azoury et al., 2015), poly(ADP-ribose) polymerase (Anders et al., 2010), mTOR inhibitors (Xie et al., 2016), hormone receptors (Ko and Balk, 2004), proteasomal components (Kisselev et al., 2012), ganglioside GD2 (Suzuki and Cheung, 2015), and cancer-specific fusion proteins (Giles et al., 2005). For many cancers, the emergence of target drugs was highly beneficial. For example, trastuzumab (anti-HER2 monoclonal antibody) and other related medications at least doubled median survival time in patients with metastatic HER2-positive breast cancer (Hudis, 2007; Nahta and Esteva, 2007). In melanoma, immune checkpoint inhibitors, and anti-BRAF target drugs like Vemurafenib and Dabrafenib dramatically increased the patient's chances to respond to treatment and to increase survival (Chapman et al., 2011; Prieto et al., 2012). Target drugs were also of a great advantage for inoperable kidney cancer, before almost uncurable (Ghidini et al., 2017).

The efficiencies of target drugs vary from patient to patient (Ma and Lu, 2011) and the results of clinical trials clearly evidence that the drugs considered inefficient for an overall cohort of a given cancer type, may be beneficial for a small fraction of the patients (Zappa and Mousa, 2016). For example, the anti-EGFR drugs gefitinib and erlotinib showed little advantage in the randomized trials on patients with non-small cell lung cancer. However, ~10-15% of the patients responded to the treatment and had longer survival characteristics. It was further understood that these patients had activating mutations of *EGFR* gene and that these mutations, therefore, can predict response to the EGFR-targeting therapies (Gridelli et al., 2011). Interestingly, the same approach was ineffective in colorectal cancer, where EGFR-mutated status had no predictive power for the anti-EGFR drugs cetuximab and panitumumab. In the latter case, it is the wild-type status of *KRAS* gene (~60% of all the cases) that is indicative of tumor response to these drugs (Grothey and Lenz, 2012).

The price for inefficient treatment is high as it is converted from decreased patient's survival characteristics and overall clinical expenses. There are currently more than 200 different anticancer target drugs approved in different countries, and this number grows every year (Law et al., 2014). However, the predictive molecular diagnostic tests are available for only a minor fraction of drugs, in a minor fraction of cancer types (Hornberger et al., 2005; Le Tourneau et al., 2014; Buzdin et al., 2018). This makes the clinician's decision on drug prescription a difficult task somewhat similar to finding needle in a haystack. The problem of choosing the right medication for the right patient is currently well understood, so US FDA(Food and Drug Administration) strongly recommends any new target drug emerging on the market to be supplied with the companion diagnostics test^1^. It is, therefore, of a great importance to identify robust predictive biomarkers of target drug efficacy, for as many cancer-drug combinations as possible. Recently, a new generation of molecular markers has been proposed involving gene combinations and even entire molecular pathways (Gu et al., 2011; Li et al., 2014; Toren and Zoubeidi, 2014). Here, the biomarkers used are not just a single gene or single locus-based mutation, expression or epigenetic features, but rather the aggregated combinations of those, crosslinking the physiologically relevant gene products (Diamandis, 2014; Sanchez-Vega et al., 2018; Zaim et al., 2018). The pathway-based approach has been better developed for the high throughput gene expression data (Khatri et al., 2012; Buzdin A. A. et al., 2017; Buzdin et al., 2018) where the *Pathway Activation Strength (PAS*) may be used as an aggregated biomarker (Buzdin et al., 2014). The formulas for *PAS* calculation may be different; they normally consider relative concentrations of gene products, internal molecular architecture of pathways and gene coexpression patterns (Ozerov et al., 2016; Aliper et al., 2017; Buzdin et al., 2018). *PAS* was shown to be more efficient as a biomarker than the individual gene expression data (Borisov et al., 2014, 2017), and *PAS* biomarkers were further generated for a plethora of normal and pathological conditions, including cancer response to treatments (Kurz et al., 2017; Petrov et al., 2017; Spirin et al., 2017; Wirsching et al., 2017; Sorokin et al., 2018).

Furthermore, a method for ranking of more than a 100 of target anticancer drugs has been recently published based on the *PAS* scoring and the pathway enrichments by the molecular targets of drugs (Artemov et al., 2015). This approach termed *Drug Scoring* was experimentally shown promising for drugs prescription to advanced solid tumor patients (Buzdin A. et al., 2017; Buzdin et al., 2018; Poddubskaya et al., 2018). However, good quality expression profiles required for *PAS*-based *Drug Scoring* frequently cannot be obtained due to apparent lack of biopsy biomaterials and RNA degradation. To our knowledge, so far there were no published reports on the application of gene mutation data for *Drug Scoring*.

In this study, for the first time we proposed and tested 10 alternative pathway-based *Drug Scoring* algorithms utilizing mutations data. These algorithms were used for the data from 3,800 published cancer mutation profiles representing eight tumor localizations and validated using the published clinical trials data. We showed that several mutation-based *Drug Scoring* methods can be used efficiently for predicting the effectiveness of target drugs. This has been evidenced by statistically significant positive correlations between *Drug Score* ratings of individual drugs and their therapeutic success reflected by the completed phases of clinical trials for the respective cancer types. We also used the best *Drug Scoring* algorithm to simulate all known protein coding genes as the potential drug targets. We found that the algorithm-predicted most efficient targets are highly congruent with the molecular targets already used by the real anticancer drugs.

## Materials and Methods

### Mutation Data

The human mutation dataset was obtained from the Catalog Of Somatic Mutations In Cancer (COSMIC) (Forbes et al., 2017). COSMIC aggregates and annotates mutation data from various sources by providing lists of verified somatic mutations. We downloaded the data from COSMIC website, version 76. The complete dataset includes 6,651,236 somatic mutation records for 20,528 genes in 19,434 tumor samples of 37 primary localizations.

### The Algorithm Validation Dataset

For the validation of drug scoring algorithms, we extracted mutation data only for the primary localizations containing at least 100 samples indexed in COSMIC and originally taken from The Cancer Genome Atlas (TCGA) project (Tomczak et al., 2015; Forbes et al., 2017) because of the uniform sequencing and data processing pipeline used there. For the algorithm validation dataset, we totally took 3,800 tumor mutation profiles from eight primary localizations: central nervous system, kidney, large intestine (including cecum, colon, and rectum), liver, lung, ovary, stomach, thyroid gland (Table 1).

**Table 1:** The structure of algorithm validation dataset.

**Localization (COSMIC nomenclature)**	**Number of samples**	**Disease, its abbreviation**
Central nervous system	657	Gliomas, GL
Kidney	601	Kidney cancer, KC
Large intestine	620	Colorectal cancer, CRC
Liver	188	Hepatic cancer, HC
Lung	569	Non-small cell lung cancer, NSCLC
Ovary	474	Ovarian cancer, OVC
Stomach	288	Stomach cancer, STC
Thyroid	403	Thyroid cancer, THC

The COSMIC data were processed with script written in R (version 3.4.3) to obtain mutation profile for each tumor^1^. The processed data is available as Supplementary Data Sheet 1.

### The Dataset for Prediction of Potential Molecular Targets

We used the full COSMIC dataset to increase the statistical significance and to investigate the effectiveness of potential target drugs for a maximum range of cancer localizations. However, we excluded the samples related to cell cultures or tumor xenograft to standardize the analysis. We excluded records having the following marks in the “Sample source” field: organoid culture, short-term culture, cell-line, xenograft. Thus, the final dataset included 6,027,881 mutations records in 18,273 in tumor samples of 35 primary localizations. The COSMIC data were processed with script written in R (version 3.4.3) to return mutation rates for all genes^2^. The processed data is available as Supplementary Data Sheet 2.

### Clinical Trials Data

We extracted clinical trials data from the web sites of NIH (the National Institutes of Health)^3^ and US FDA^4^. They were processed by manually curation of web data as of July 2017. The processed clinical trials data used for the correlation studies are shown on Supplementary Table 1.

### Molecular Pathways Data

The gene contents data about 3,125 human molecular pathways used to calculate mutation drug scores were extracted from Reactome (Croft et al., 2014), NCI Pathway Interaction Database (Schaefer et al., 2009), Kyoto Encyclopedia of Genes and Genomes (Kanehisa and Goto, 2000), HumanCyc (Romero et al., 2004), Biocarta (Nishimura, 2001), Qiagen^5^. For drug scores calculation, we used only the 1,752 pathways including at least 10 gene products because of previously reported poor theoretical data aggregation effect for smaller pathways (Borisov et al., 2017). The information about molecular specificities of 128 anticancer target drugs were obtained from databases DrugBank (Law et al., 2014) and ConnectivityMap (Lamb et al., 2006).

### Data Presentation

The results were visualized using package ggplot2 (Wickham, 2009).

## Results

In this study, we developed a molecular pathway-based method of target drug scoring using high throughput mutation data.

### Algorithms of Mutation Drug Scoring

The principle of *Mutation Drug Scoring (MDS)* methods proposed here deals with quantization of mutation enrichment for the molecular pathways having molecular targets of a drug under investigation. Overall, they are based on the rationale that the greater is the mutation level of the respective pathways, the higher will be the expected drug efficiency. The mutation enrichment of a molecular pathway called pathway instability (*PI*) is calculated based on the relative *mutation rates* (*MR*) of its member genes. Under *mutations*, we meant here the changes in protein coding sequence understood as such in the Catalog of Somatic Mutations in Cancer (COSMIC) v.76 database (Forbes et al., 2016). COSMIC is the world's largest database of somatic mutations relating to human cancers. We used only Genome-wide Screen Data to estimate *MR* correctly. This part of COSMIC consists of peer reviewed large-scale genome screening data and data from the validated sources such as The Cancer Genome Atlas (TCGA) and International Cancer Genome Consortium (ICGC).

*Mutation rate* (*MR*) is calculated according to the formula:

$$MR_{n,g}=\frac{N\;mut(\;n,g)}{N\;samples\;(g)},$$

where *MR*_*n, g*_ is *MR* of a gene *n* in a group of samples *g*; *N mut(n,g)* is the total number of mutations for gene *n* in a group of samples *g*; *N samples (g)* is the number of samples in a group *g*. The *MR* values strongly positively correlated with the lengths of gene coding DNA sequence (CDS; data not shown). In order to remove bias linked with the CDS length, we took for further consideration a normalized value termed *Normalized Mutation Rate* (*nMR*) expressed by the formula:

$$nMR_{n}=\frac{1000*MR_{n}}{Length\;CDS\;(n)\;},$$

where *nMR*_*n*_ is the *nMR* of a gene *n*; *MR*_*n*_ is the *MR* of a gene *n*; *Length CDS(n)* is the length of CDS of gene *n* in nucleotides. Indeed, normalization of this metric enabled to terminate any CDS-linked bias (data not shown).

To determine if gene *n* is included in pathway *p*, we introduced a Boolean flag *pathway-gene indicator PG*_*n*__, p_ expressed by the formula:

$$PG_{n,p}=\;\{\begin{array}{c} 1,\text{ pathway }p\text{ includes gene }n,\\ 0,\text{ pathway }p\;{\text{doesn}}^{'}\text{t include gene n}; \end{array}$$

The *Pathway Instability* (*PI*) score is then calculated as follows:

$$PI_{p}=\sum_{n}nMR_{n}PG_{n,p},$$

where *PI*_*p*_ is pathway instability score for a pathway *p*; *nMR*_*n*_ is the *normalized* mutation rate of a gene *n*, *PG*_*n*__, p_ is pathway-gene indicator for gene *n* and pathway *p*. *Pathway instability score* characterizes the mutation enrichment of a pathway (Pathway instability is an effective new mutation-based type of cancer biomarkers, 2018, in preparation). To formalize if gene *n* is molecular target of drug *d*, we introduced another Boolean flag *drug target index*, *DTI*_*d, n*_:

$${\text{DTI}}_{\text{d},\text{n}}=\{\begin{array}{c} 1,\text{ drug }d\text{ has target gene }n,\\ 0,\text{ drug }d\;{\text{doesn}}^{'}\text{t have target gene }n \end{array}$$

To complete *DTI* database for this study, we used the data about molecular specificities of 128 target drugs extracted from the databases DrugBank (Law et al., 2014) and Connectivity Map (Lamb et al., 2006).

To link *PI* scores and estimated drug efficiencies, the following basic formula was proposed for the calculation of *Mutation Drug Score* (*MDS*):

(1) $$\text{MD}{\text{S}}_{\text{d}}=\sum_{\text{n}}\text{DT}{\text{I}}_{\text{d},\text{n}}\sum_{p}PG_{n,p}PI_{p},$$

where *d* is drug name; *n* is gene name; *p* is pathway name; *MDS*_*d*_ is *MDS* for drug *d*; *DTI*_*d, n*_ is drug target index for drug *d* and gene *n*; *PI*_*p*_ is *Pathway Instability* of pathway *p*; *PG*_*n*__, p_ is pathway-gene indicator for gene *n* and pathway *p*.

The above basic formula (*1*) was modified to generate several alternative methods of drug scoring.

-*Pathway size-normalized*. Since molecular pathways include considerably different number of genes varying from dozens to hundreds, we proposed a modification of the calculation method (*1*) where normalization is performed for *MDS* on the respective number of genes for each *PI* member:
  (2) $$MDS\_N_{d}=\sum_{n}\text{DT}{\text{I}}_{\text{d},\text{n}}\sum_{p}PG_{n,p}PI_{p}/k_{p},$$
  where *k*_*p*_ is number of genes in pathway *p*.
- *Single count-normalized*. Impact of each gene participating in pathways targeted by drug *d* is counted only once:
  (3) $$\text{MDS}\_\text{gen}{\text{e}}_{\text{d}}=\sum_{n}\text{nM}{\text{R}}_{n}\;\text{GI}{\text{I}}_{d,n},$$
  where *GII*_*d, n*_ – Boolean flag *gene involvement index*,
  $$\begin{array}{c} {\text{GII}}_{d,n}\text{=}\\ \{\begin{array}{c} 1,\text{ gene }n\text{ participates in at least one pathway targeted by drug }d\\ 0,\text{ gene }n\;{\text{doesn}}^{'}\text{t participate in pathways targeted by drug }d \end{array} \end{array}$$
- *Number of pathways-normalized*. *MDS* for drug *d* is normalized on the number of its targeted molecular pathways.
  (4) $$\text{MDS}\_{\text{m}}_{\text{d}}=MDS_{d}/m_{\text{d}},$$
  where *m*_*d*_ – number of pathways targeted by drug *d*.
- *Number of pathways-normalized*. *MDS_N* is additionally normalized on the number of pathways targeted by drug *d (m*_*d*_*)*.
  (5) $$\text{MDS}\_\text{N}\_{\text{m}}_{\text{d}}=MDS\_N/m_{d}$$
- *Number of target genes-normalized*. *MDS_b*_*d*_ is additionally normalized on the number of target genes for drug *d, (b*_*d*_).
  (6) $$\text{MDS}\_{\text{b}}_{\text{d}}=MDS_{d}/b_{d}$$
- *Number of target genes-normalized MDS_N*. *MDS_N*, normalized on the number of target genes for drug *d, (b*_*d*_).
  (7) $$\text{MDS}\_\text{N}\_{\text{b}}_{\text{d}}=\text{MDS}\_\text{N}/b_{d}$$
- *Number of target genes-normalized MDS_gene*. *MDS_gene*, normalized on the number of target genes for drug *d, (b*_*d*_).
  (8) $$\text{MDS}\_\text{gene}\_{\text{b}}_{\text{d}}=MDS\_gene/b_{d}$$
- *Target genes dependent only. MDS2* is calculated considering only mutation frequencies of target genes.
  (9) $$\text{MDS}2_{\text{d}}=\sum_{p}PG_{n,p}\;\sum_{n}\text{DT}{\text{I}}_{\text{d},\text{n}}nMR_{n}$$
- *Single count-normalized, target genes dependent only. MDS2_gene* is calculated, considering each target gene for drug *d* only one time.
  (10) $$\begin{array}{l} \text{MDS}2\_\text{gen}{\text{e}}_{\text{d}}=\sum_{n}\text{DT}{\text{I}}_{\text{d},\text{n}}\;NMR_{n}\;\text{GI}{\text{I}}_{d,n} \end{array}$$
  For these algorithms of mutation-based drug scoring, we next compared their congruences with the published clinical trials data.

### Validation of Mutation Drug Scoring (MDS) Algorithms on Clinical Trials Data

We calculated different versions of *MDS* according to formulae (1–10) for 128 anticancer target drugs, for eight cancer types (Supplementary Data Sheet 3). We examined somatic mutation profiles for 3,800 samples of the following primary tumor localizations: large intestine (including cecum, colon and rectum), lung, kidney, stomach, ovarian, central nervous system, liver, thyroid (Table 1).

Mutation profiles were extracted from COSMIC v76 database (Forbes et al., 2016). To validate the *MDS* algorithms, we selected only tumor samples related to TCGA project because it was the largest source of biosamples profiled using a single deep sequencing and bionformatic pipeline (Tomczak et al., 2015). Molecular specificities of drugs were obtained from DrugBank (Law et al., 2014) and Connectivity Map (Lamb et al., 2006) databases. The information about clinical approval and the completion of phases of clinical trials for 128 target drugs for the above eight tumor localizations was taken from the web sites of NIH and US FDA. To measure completion of clinical investigations for a drug, we introduced the metric termed *Clinical Status*. These values are congruent with the apparent efficiencies of drugs for the given cancer types. The same drugs most frequently had different clinical statuses for the different cancer types.

The *Clinical Status* varied in a range from 0 to 1 proportional to the top phase of clinical trials passed by a drug for a given cancer type. The *Clinical Status* grows incrementally depending on the completion of the clinical trials phases 1–4, while the later phases have a greater specific weight, because they allow to more accurately determine clinical efficacy of a drug (Table 2).

**Table 2:** Clinical Status of drug, according of the top passed phases of clinical trials.

**Phase of clinical trials**	**Clinical status**
Phase I ongoing	0.1
Phase I/II ongoing (Phase I completed)	0.2
Phase II ongoing	0.3
Phase II completed	0.4
Phase III ongoing	0.7
Phase III completed	0.85
Phase IV (drug approved and marketed)	1

The complete *Clinical Status* information for 128 drugs under investigation is shown on Supplementary Table 1. The major limitation of this approach is that only the drugs that had been already clinically investigated for the respective tumor type can be ranked in such a way.

**Figure 1:**
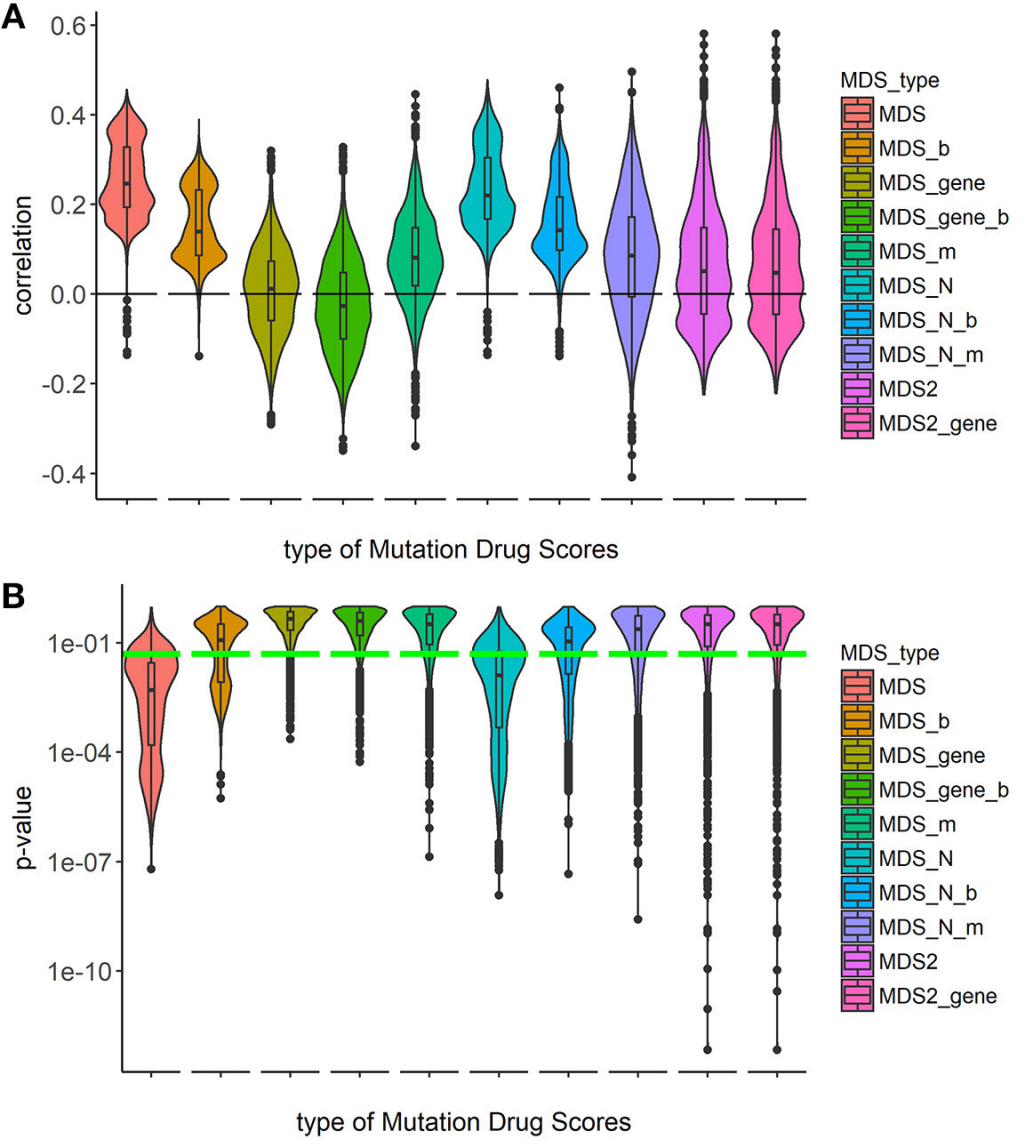
Correlation between Clinical Status and MDS rank for 10 types of drug scoring in eight cancer types at once. **(A)** Distributions of Spearman correlation coefficients between Clinical Status and MDS rank for 128 target drugs in 3,800 tumor samples. MDS rank of a drug was calculated as the individual drug's position in the rating (from top to low) of all drugs under investigation. Ten violin plots distributed along X-axis, each represent a particular type of drug scoring. The Y-axis reflects density distributions of correlations between Clinical Status and MDS ranks. Boxes indicate the second and third quartiles of distribution, black dots indicate outliers. **(B)** The plot demonstrates the distributions of *p*-value for the correlation coefficients between Clinical Status and MDS rank for 128 target drugs in the same tumor samples. The horizontal green line corresponds to *p* = 0.05.

To investigate the capacities of different versions of *Mutation Drug Scores* to successfully predicts clinical efficiencies of drugs, we analyzed how ranks of *MDS* values correlated with Clinical Status of drugs. We calculated correlations and compared distributions of the Spearman correlation coefficients. To calculate correlations, we took all cancer mutation profiles together without separation on cancer types (Figure 1).

**Figure 2:**
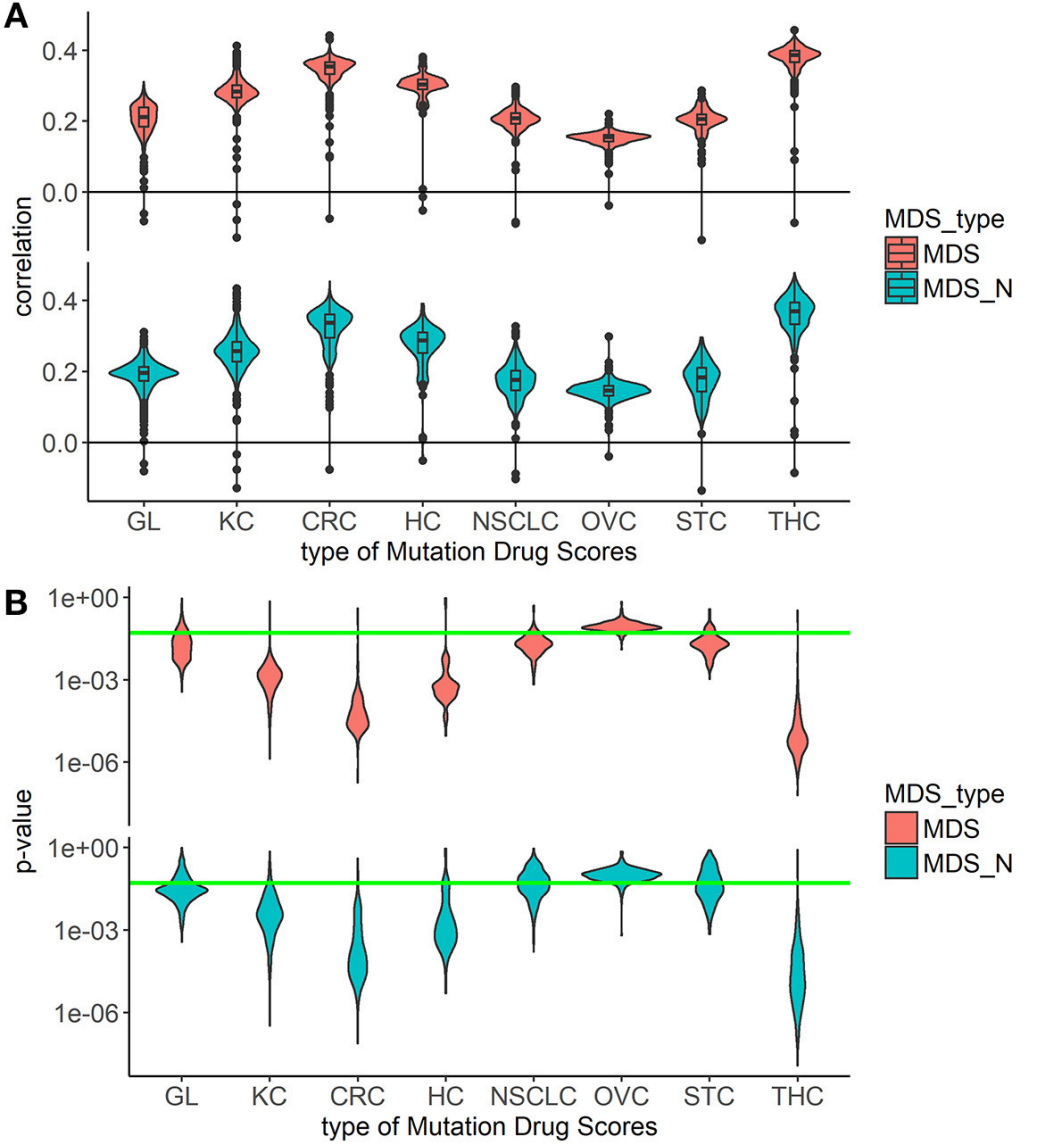
Correlation between Clinical Status and MDS rank for two best types of drug scoring in eight cancer types separately. **(A)** Distributions of Spearman correlation coefficients between Clinical Status and MDS rank for 128 target drugs in eight tumor types. MDS rank of a drug was calculated as the drug's position in the rating (from top to low) of all drugs under study. The drug scoring methods are shown in horizontal lines, and the cancer types are placed vertically. The violin plots distributed along X-axis, each represent a particular cancer type. The Y-axis reflects density distributions of correlations between Clinical Status and MDS ranks. Boxes indicate the second and third quartiles of distribution, black dots indicate outliers. **(B)** The plot shows the distributions of *p*-value for the correlation coefficients between Clinical Status and MDS rank for 128 target drugs in the same tumor types. The horizontal green line corresponds to *p* = 0.05.

Overall, the markedly better correlations were seen for the *MDS* and *MDS_N* types of drug scoring (Figure 1). We next analyzed the cancer type-specific distributions (Figure 2). It was seen that both *MDS* and *MDS_N* scores positively correlated with the drugs clinical efficiencies in all the localizations investigated, thus confirming their top status among the drug scoring algorithms. Among those, *MDS* showed best overall functional characteristics and was, therefore, used in further analyses.

### Application of MDS for Identification of Possible Target Genes

We next tested the *MDS* algorithm for its capacity to identify potentially valuable drug targets. To this end, we modeled a situation when each gene specifically corresponds to one target drug. Those simulated, or virtual drugs, also were specific each to only one gene product. Using the database of 1,752 molecular pathways, we were able to calculate *MDS* for 8,736 *virtual drugs* specific to the same number of genes included in these pathways. For this analysis, we used 18,273 full-exome tumor mutation profiles from the COSMIC v76 database. Top 30 molecular targets with highest *MDS* values and already clinically approved cancer drugs specific for these molecular targets are listed on Table 3. The complete *MDS* calculation data are given in Supplementary Table 2.

**Table 3:** Top 30 molecular targets sorted by MDS and clinically approved drugs using these molecular targets.

**Potential molecular targets**	**MDS**	**Existing relevant drugs**
PIK3CA	387.11	Idelalisib
PIK3R1	371.31	
MAPK1	354.75	
MAPK3	343.81	
HRAS	343.66	
PIK3CB	313.02	Idelalisib
AKT1	305.54	Perifosine
PIK3R2	302.74	
PIK3CD	293.15	Idelalisib
KRAS	291.42	
PIK3R3	290.07	
MAP2K1	288.80	Binimetinib, cobimetinib, selumetinib, trametinib
NRAS	287.90	
PIK3R5	279.34	
RAF1	271.72	Dabrafenib, regorafenib, sorafenib
MAPK8	267.73	
MAP2K2	257.33	Binimetinib, cobimetinib, selumetinib, trametinib
TP53	255.89	
GRB2	254.36	
SOS1	243.39	
RAC1	239.32	
MAPK9	233.01	
EGFR	232.80	Afatinib, brigatinib, cetuximab, erlotinib, flavopiridol, foretinib, gefitinib, lapatinib, masitinib, nimotuzumab, osimertinib, panitumumab, vandetanib, necitumumab
MAPK14	224.08	
MAPK10	222.51	
EGF	214.20	
RELA	212.43	
PRKCA	211.99	
NFKB1	211.63	Thalidomide
AKT2	205.38	Perifosine

**Figure 3:**
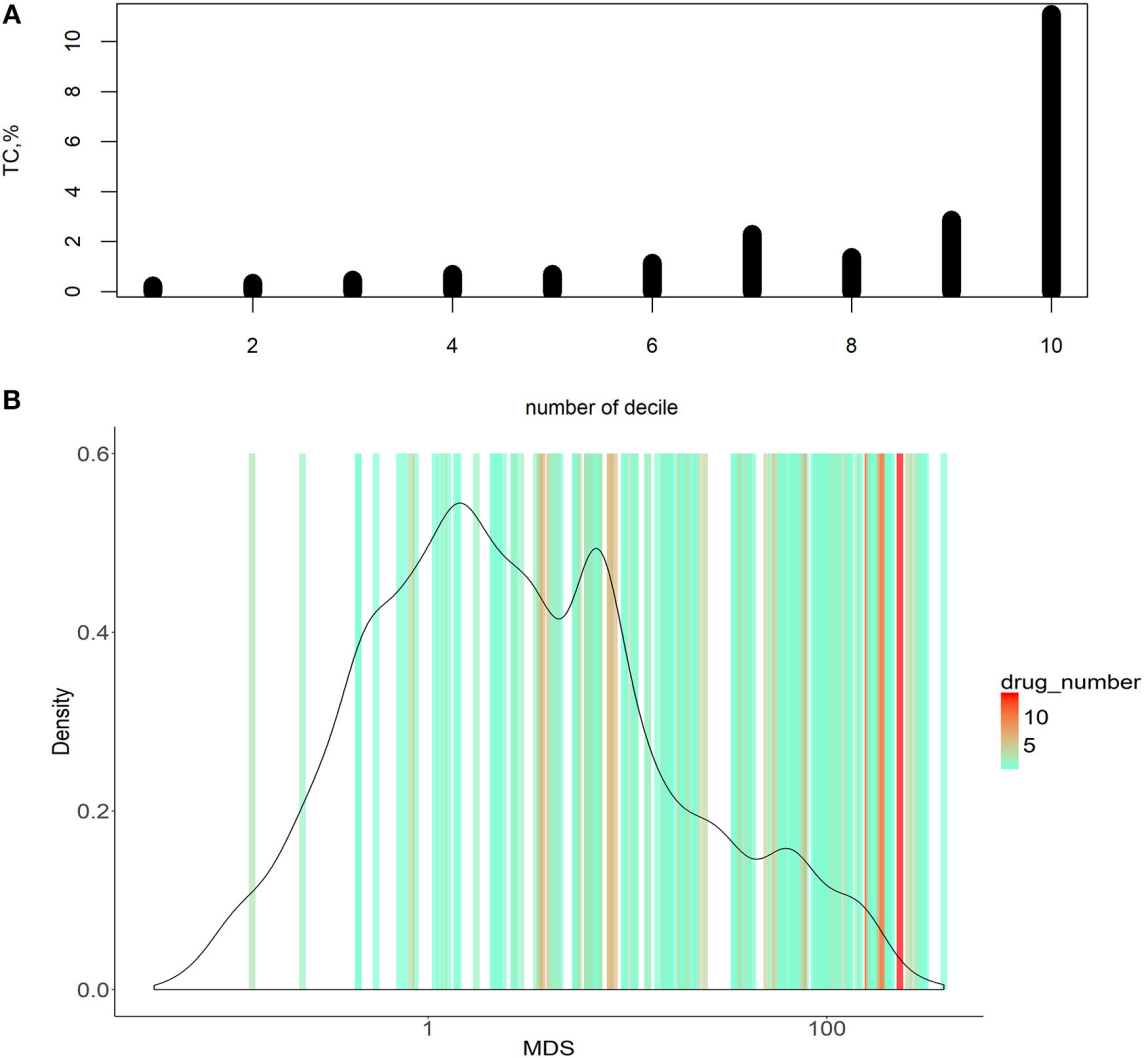
Dependence of MDS and occurrence of molecular targets in approved cancer drugs. **(A)** Deciles of potential molecular targets sorted in ascending order according to MDS value. TC was calculated for each decile, shown on vertical axes. **(B)** Distribution of MDS values among the potential molecular drug targets. The color scale on the graph indicates densities of clinically approved cancer drugs exploiting the respective molecular targets.

We next ranked all the *virtual* drugs according to their *MDS* values and compared if the same molecular targets are already exploited by the *existing* 128 target cancer drugs (Figure 3).

To do this, we introduced an auxiliary value termed *Target Conversion* (*TC*). It reflects the percentage share of *known* molecular targets among *predicted* molecular targets.

$$TC=\frac{number\;of\;known\;molecular\;targets\;}{number\;of\;predicted\;molecular\;targets}*100\%$$

For the overall (complete) list of potential molecular targets, *TC* was 2.17%. However, there was an clear-cut incremental *TC* growth trend when the potential molecular targets were sorted in the ascending order of *MDS* value (Figure 3A, shown for deciles of the potential targets). The greater *TC* value exceeding 10% was observed for the decile of molecular targets having the highest *MDS* values.

Molecular targets with the highest *MDS* are clearly enriched by the existing clinically approved drugs compared to those with low *MDS* scores (Figure 3A). On the other hand, target genes with higher *MDS* are covered by a bigger number of approved drugs per target, as many drugs have common molecular specificities (Figure 3B).

**Figure 4:**
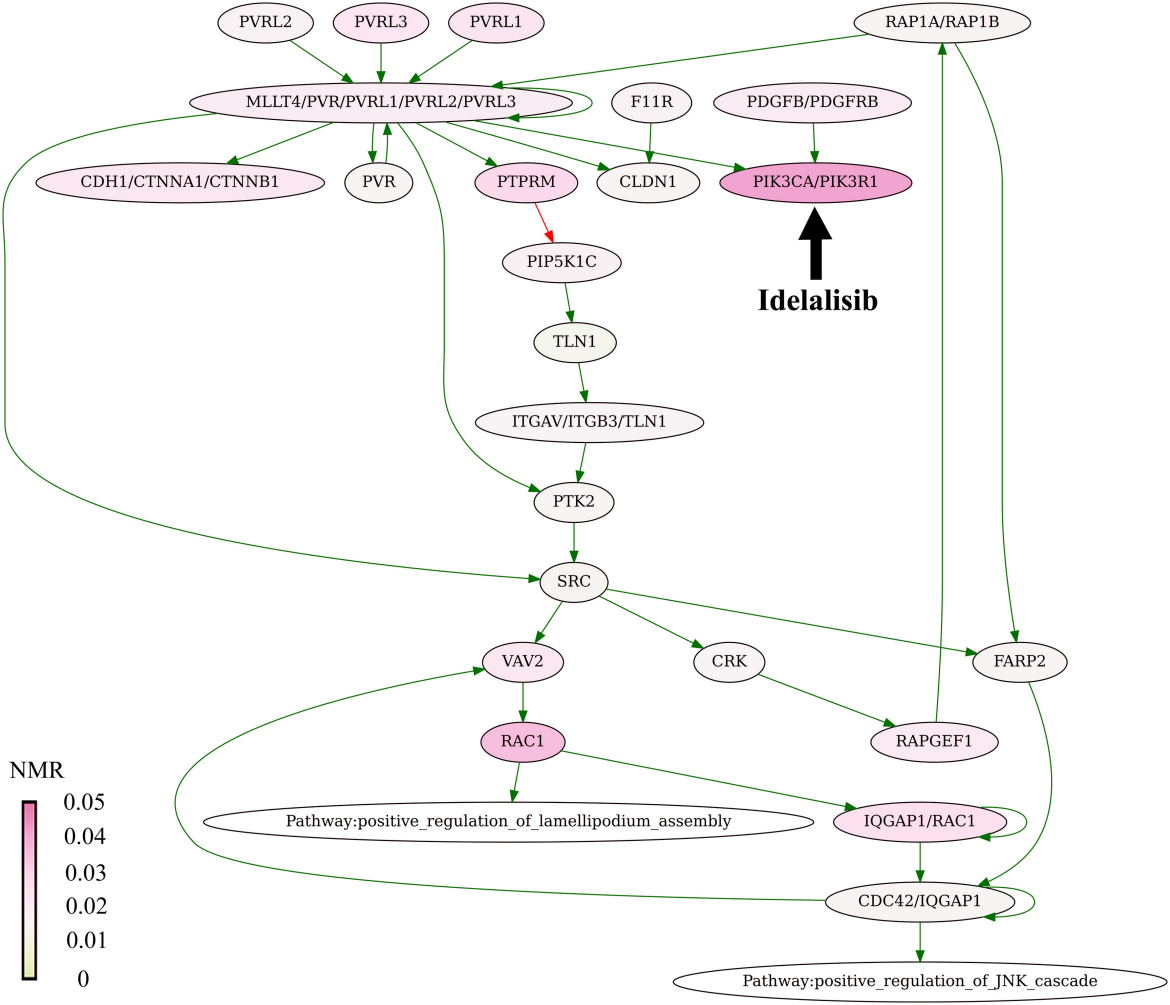
Mutation enrichment of Nectin adhesion pathway. The pathway is targeted by Idelalisib. The pathway structure is taken from the NCI database (Schaefer et al., 2009). The mutation burden was visualized using Oncobox pathway plot tool. The color scale reflects mutation levels of the corresponding nodes on the pathway graph. The green arrows indicate activation, red arrow—inhibition, bold black arrow indicates molecular target of Idelalisib.

The present algorithm for scoring potential drug targets considers a cumulative mutation enrichment of molecular pathways. For the example shown on Figure 4 (Nectin adhesion pathway), most genes involved in a pathway are mutated in cancers, see the color scale. The mutation enrichment of a pathway may characterize its overall involvement in malignization. According to the present conception of drug scoring, the maximum efficiency of drug can be obtained by acting on the most strongly affected molecular pathways.

## Discussion

In this study, we report a new bioinformatic instrument of ranking target anticancer drugs using high throughput gene mutation data. We proposed here 10 different versions of molecular pathway-based *mutation drug scoring*. At least two types of this scoring could provide output data positively correlated with the clinical trials data for 128 drugs in all eight tumor localizations tested. We hope that the pathway-based mutation drug scoring approach has a potential of helping clinical oncologists to implement personalized selection of target drugs based on the individual, the patient's tumor-specific high throughput mutation profile.

We showed that the same approach can be applied to identify potentially efficient molecular targets in experimental oncology. The educated choice of new drug targets is one of the main tasks in pharmacology (Schenone et al., 2013). Experimental search for new efficient drug targets is still time consuming, laborious, and expensive (Haggarty et al., 2003), so since recently a credit is frequently given to computational predictive algorithms (Rifaioglu et al., 2018).

The history of computational prediction of drug targets began with prediction of druggability based on the structure of targets and biomedical text mining (Cheng et al., 2007; Zhu et al., 2007). Several methods have been also proposed based on known links between drugs and genes (Luo et al., 2017). Further development of bioinformatic methods allowed to apply for this task a set of systems approaches based on networks of molecular interactions (Mani et al., 2008).

Our results provide principal evidence that the mutation drug scoring is applicable to ranking of anticancer drugs. On the other hand, our data suggest that these drug scoring algorithms can be applied for the identification of novel molecular targets for the prospective anticancer drugs. Although many genes with high *MDS* already serve as molecular targets of the approved cancer drugs, there is a number of top *MDS* genes that are not yet covered by the existing medications. This latter fraction of genes, therefore, can be considered a source of potential targets for new drug developments. For example, the following top 100 *MDS* genes can be mentioned that are not yet covered by approved or experimental cancer or antineoplastic drugs [according to DrugBank (Law et al., 2014), DGIdb (Cotto et al., 2018), FDA^6^, HMDB (Wishart et al., 2018), Tocris^7^, GeneCards (Safran et al., 2010) databases]: *GRB2, SOS1,SOS2, SHC1, GNB1, CREB1, GNG2, GNAQ, GNB5, GNAI2. Three* of them (*GRB2, GNG2, CREB1*) are the targets of approved non-oncological drugs **(**Pegademase bovine, Naloxone, Adenosine monophosphate, Citalopram, Halothane**)**, thus illustrating *MDS* method potential in drug repurposing.

This study can be regarded as proof-of concept trial of *MDS* approach exemplified by bigger proportion of real cancer targets among the genes with higher *MDS* values. In this application, we assessed integral *MDS* for all cancer types. However, in further applications the same approach can be used for any specific tumor type or subtype to identify targets that may seem most promising for this particular disease. This could be valuable, for example, for drugs repurposing among the different tumor types and for more effectively identifying the patient cohorts in clinical trials

The present mutation drug scoring approach scores the molecular pathway instability caused by accumulation of mutations and ranks drugs according to a simple rationale—the higher is mutation burden of a pathway, the greater may be the efficiency of a drug targeting this pathway. We hope these findings will be interesting to those working in the fields of oncology, drug discovery, systems biomedicine, high throughput mutation data analysis, personalized medicine and molecular diagnostics.

## Data Availability Statement

The datasets analyzed for this study can be found in the COSMIC repository (COSMICv76; CosmicGenomeScreensMutantExport. tsv.gz, https://cancer.sanger.ac.uk/cosmic/download).

## Author Contributions

MZ developed algorithms, did mutation drug scoring analyses, planned the research and wrote the manuscript. PB planned the research, extracted and filtered cancer mutation data. AG planned the research and developed algorithms. MS completed the molecular pathway database. DK completed and processed Clinical Status database. AE organized the information about molecular specificities of anticancer target drugs. NB developed algorithms, did statistical analyses, and planned the research. AB completed Clinical Status database, developed algorithms, planned the research, and wrote the manuscript.

### Conflict of Interest Statement

MS, NB, AG were employed by company Omicsway Corp. MZ, AB were employed by company Oncobox Ltd. The remaining authors declare that the research was conducted in the absence of any commercial or financial relationships that could be construed as a potential conflict of interest.

## Footnotes

^1^For Consumers - Personalized Medicine and Companion Diagnostics Go Hand-in-Hand Available at: https://www.fda.gov/ForConsumers/ucm407328.htm [Accessed October 15, 2018].

^2^Cosmic v76 processing Available at: https://gitlab.com/White_Knight/cosmic76_processing/tree/master [Accessed October 22, 2018].

^3^ClinicalTrials.gov Available at: https://clinicaltrials.gov/ [Accessed July 25, 2017]

^4^U S Food and Drug Administration Home Page Available at: https://www.fda.gov/ [Accessed July 25, 2017].

^5^QIAGEN - Sample to Insight Available at: https://www.qiagen.com/us/shop/genes-and-pathways/pathway-central/ [Accessed September 19, 2018].

^6^U S Food and Drug Administration Home Page Available at: https://www.fda.gov/ [Accessed July 25, 2017].

^7^Tocris Bioscience Available at: https://www.tocris.com/ [Accessed December 21, 2018].

## Supplementary Material

Supplementary Data Sheet 1 Mutation profiles for 3800 TCGA tumor samples obtained from COSMIC v76.

Supplementary Data Sheet 2 Normalized mutation rate for genes that mutated in 18273 tumor samples from COSMIC v76.

Supplementary Data Sheet 3 Mutation drug scores for 3800 TCGA tumor samples for 10 versions of drug scoring algorithm. The dataset contains 10 tables that match the 10 methods of drug scoring.

Supplementary Table 1 The complete clinical status information for 128 target drugs in eight cancer localizations.

Supplementary Table 2 Mutation Drug Scores for 8736 virtual drugs specific to the same number of genes.

## Abbreviations

CDS length: Coding DNA Sequence Length
COSMIC: Catalog Of Somatic Mutations In Cancer
FDA: Food and Drug Administration
ICGC: International Cancer Genome Consortium
MDS: Mutational Drug Scores
MR: Mutation rate
nMR: Normalized mutation rate
NIH: The National Institutes of Health
PAS: Pathway Activation Strength
PI: Pathway instability
TCGA: The Cancer Genome Atlas
TC: Target Conversion.
